# The unmet need of organ transplantation in Africa

**DOI:** 10.1097/JS9.0000000000000025

**Published:** 2023-03-24

**Authors:** Wireko A. Awuah, Jyi Cheng Ng, Halil I. Bulut, Abubakar Nazir, Pearl O. Tenkorang, Rohan Yarlagadda, Mubarak J. Mustapha, Toufik Abdul-Rahman, Aymar Akilimali, Victor N. Oti, Narjiss Aji

**Affiliations:** a Sumy State University, Sumy, Ukraine; b Faculty of Medicine and Health Sciences, University of Putra Malaysia, Serdang, Malaysia; c Istanbul University Cerrahpasa, Cerrahpasa School of Medicine, Istanbul, Turkey; d King Edward Medical University, Lahore, Pakistan; e University of Ghana Medical School, Accra, Ghana; f Rowan University School of Osteopathic Medicine, Stratford, New Jersey, USA; g Faculty of Basic Medical Sciences, University of Ilorin, Ilorin, Nigeria; h Faculty of Medicine, Official University of Bukavu, Bukavu, Democratic Republic of the Congo; i Kyiv Medical University, Polish Campus, Ukraine; j Faculty of Medicine and Pharmacy of Rabat, Mohamed V University, Rabat, Morocco

*Dear Editor*,

Africa, a promising continent with a population of over 1.2 billion people, faces a massive disease burden due to their fragile healthcare systems. The prevalence of diseases in Africa, particularly noncommunicable diseases is rapidly increasing and, if not controlled, can have a negative impact on the continent, given the challenges Africa already faces. Noncommunicable diseases cause most end-stage organ failures worldwide and are the leading cause of death, accounting for 71% of global deaths and 85% of deaths in low- and middle-income countries^1^. Organ transplantation, one of modern medicine’s breakthroughs, has been clinically successful in treating end-stage organ failure. Unfortunately, just a few states are offering organ transplants in Africa, which include Ivory Coast, South Africa, Seychelles, Sudan, Nigeria, Namibia, Kenya, Ethiopia, Ghana, Mauritius, and Tanzania. Surprisingly, only 643 organ transplants were recorded in Africa in 2016^1^.

Although Africa lags behind other regions in terms of transplantation capacity, there has been significant progress in the past decades. Initiatives have been implemented to boost the understanding of transplant surgeries. For instance, in 2013, South Africa invited 10 countries (Cameroon, Ethiopia, Ghana, Kenya, Malawi, Nigeria, Rwanda, Senegal, Sudan, Tunisia, and Zambia) for a Global Alliance on Transplantation meeting with the aim of equipping them with the needed skills and knowledge for organ transplantation^2^. Also, the creation of an acceptable declaration where organ donation and transplantation programs can function depended heavily on government backing. While numerous participating countries identified that there are no satisfying or detailed regulations in place, Ghana and Nigeria indicated, however, that medical practitioners are presently advocating authorities to enact transplantation laws^2^. In Nigeria, there are progressive efforts made by surgeons to establish a liver transplant center in their hospital. One of these initiatives includes a yearly conference which explores obstacles each doctor faces when doing transplants and to devise a strategy for overcoming those obstacles^3^. To establish a transplantation registry and train its staff in all liver transplant specialists, the same hospital has started a bilateral partnership with a liver transplant center in Malaysia and Turkey^3^.

In the context of global efforts, there are resolutions made by the World Health Assembly (WHA) and guidelines by the WHO to regulate all forms of transplantation. The WHO has set up a task force to ensure proper care is delivered globally which will benefit Africa as well^1^.

Nevertheless, Africa faces major setbacks in organ transplant services. An example of these setbacks is insufficient transplant facilities. For example, only seven African states including Algeria, Côte d’Ivoire, Ethiopia, Kenya, Namibia, Nigeria, and Uganda have facilities for kidney transplantation. In fact, the entire African continent has just 35 centers for kidney transplantation. Uganda and Namibia have only one heart transplant center each. Tissue typing, cross-matching, and some viral studies, which are major aspects of patient preparation, are mostly done overseas delaying the procedure and increasing the cost of transplantation. Adequate histological evaluation of biopsy specimens is largely unavailable, making prompt management of rejections and infections problematic^2^.

Besides, poor infrastructure, lack of institutional support and technical skills like human resources aggravate the challenges^3^. Lack of financial protection and insufficient national and international funding halt the progress in organ transplantation and availability of immunosuppressive therapies^3^. For example, only Algeria provides facilities of free immunosuppressive therapies to the recipients. The inflated cost of organ transplants and postoperative therapies along with insufficient financial support in many states contribute to the challenges associated with organ transplantation^3^.

Skilled workforce is the cornerstone of the healthcare system of every country. Organ transplantation includes close coordination of health professionals including transplant surgeons, nephrologists, pathologists, and skilled nurses. In African countries, there is an extreme shortage of healthcare workers. There are only 2.3 healthcare workers for every 1000 persons available in Africa, whereas developed countries like America have 24.8 healthcare workers per 1000 individuals^4^. Of 47 countries in sub-Saharan Africa, only 15 countries have data regarding nephrologists. There is a paucity of data regarding the number of surgeons, particularly transplant surgeons on the African continent. Lack of opportunities, uncertainty in employment, and poor financial conditions have resulted in brain drain of African highly skilled workers to the developed countries^5^.

The global increase in organ failure has spiralled the demand for organ donors, particularly in Africa. Given the enormous burden of organ failure on the African continent already, as well as the medical management costs associated with this burden, organ transplantation is both economically and medically necessary. However, as in the rest of the world, there is a scarcity of donors, particularly cadaver donors in Africa. For example, only three countries in sub-Saharan Africa; Nigeria, Kenya, and South Africa, have a functioning kidney transplant program with only South Africa performing cadaver transplants^6^. Religious and cultural beliefs play a significant role in Africa’s donor shortage. There are superstitions about organ transplantation, which can only be corrected through awareness and enlightenment campaigns^7^. These sociocultural beliefs impede organ harvesting from people who have been medically declared to be dying. Other than that, the problem of illiteracy is more common in Africa, limiting the awareness and education on the potential possibilities of organ donation^8^. As a result, we can summarize the donor problems as a lack of public awareness and education on organ donation, widespread infectious diseases, and lack of active transplant centers.

Due to the poor situation with organ transplantation in Africa, effective measures have to be taken to combat issues faced with organ transplantation to strengthen the healthcare system and reduce disease burden. There is a need to expand the current capacity of organ transplantation services in Africa by increasing transplant centers and upgrading already existing transplant facilities and infrastructure. Pretransplantation and posttransplantation services should be established in Africa to reduce the need to rely on overseas services and to improve cost-effectiveness. African governments should also consider a national insurance scheme to reduce the cost of organ transplantation services and relieve the financial burden of their citizens.

Furthermore, Africa should also build up their organ transplantation workforce involving a multidisciplinary team which consists of transplant surgeons, transplant physicians, pharmacist, social worker, dietitian, counsellor and a clinical coordinator. Transplant education and training programs should be well-constructed to ensure the delivery of safe and effective transplantation services. International collaborations have shown to be effective and should be encouraged between African institutions and transplant centers worldwide to facilitate knowledge transfer. Nongovernmental organizations and government agencies should promote public awareness about organ donation to address the myths about organ transplantation and promote organ donation. There should be more research on the management of organ failure in Africa and implementation of transplantation services in local settings should be done.

Finally, organ transplant programs in Africa will be enhanced by following the recommendations of the 2007 WHO Regional Consultation, which call for the creation of national legal frameworks, independent organ donation, and transplantation in individual countries, open organ transplant strategies, and the avoidance of commercialized organ transplants and transplant transparency. In addition, each African country should set up its own national transplant registry in accordance with the recommendations by the WHO Regional Committee for Africa.

## Ethical approval

Not applicable.

## Sources of funding

None.

## Authors’ contribution

W.A.A. and H.I.B.: conceptualized the ideas. J.C.N., R.Y., P.O.T., and W.A.A.: reviewed and edited the manuscript. All authors were involved in data curation, writing of initial draft, and final draft.

## Conflicts of interest disclosure

The authors declare that they have no financial conflict of interest with regard to the content of this report.

## Research registration unique identifying number (UIN)

Not applicable.

## Guarantor

Wireko A. Awuah.
